# Acute 3.5-minute light-intensity exercise enhances executive function and psychological mood in children

**DOI:** 10.1038/s41598-025-27358-2

**Published:** 2025-12-05

**Authors:** Takashi Naito, Koichiro Oka, Kaori Ishii

**Affiliations:** 1 Graduate School of Sport Sciences, Waseda University, 2-579-15 Mikajima, Tokorozawa, 359–1192 Saitama Japan; 2 Organization for the Strategic Coordination of Research and Intellectual Properties, Meiji University, Jinbocho, Chiyoda-ku, 101–8301 Tokyo Japan; 3 Faculty of Sport Sciences, Waseda University, 2-579-15 Mikajima, Tokorozawa, 359–1192 Saitama Japan

**Keywords:** Cognition, fNIRS, Inhibitory control, Physical activity, Positive affect, Prefrontal cortex, Psychology, Risk factors

## Abstract

Physical inactivity and sedentary behavior among children are increasing globally, raising concerns about physical and mental health, as well as brain development. Creating effective exercise programs in everyday settings (e.g., classrooms) is essential for supporting children’s health. We examined whether brief, light-intensity exercise improves executive function (Color-word Stroop task; CWST) and mood (Two-Dimensional Mood Scale; TDMS). Thirty-one children (12.0 ± 1.1 years, 54.8% boys) completed a two-condition crossover trial: (1) control, where participants completed CWST and TDMS before and after 15-min seated rest; and (2) exercise, performing a 3.5-min light-intensity exercise during rest. A two-way repeated-measures ANOVA (Condition × Time) showed significant interactions for incongruent reaction time (F [1,30] = 8.33, η² = 0.22), pleasure (F [1,30] = 11.74, η² = 0.28), and arousal (F [1,30] = 5.91, η² = 0.17), indicating faster responses and higher pleasure after exercise versus control, with arousal maintained after exercise but declining in the control condition. However, no significant associations were observed between reaction time or pleasure improvements and prefrontal cortex activation changes. No significant interactions occurred for neutral reaction time or error rates. These findings suggest that this simple intervention could be a practical strategy for improving cognitive and emotional outcomes in schools.

**Supplementary Information:**

The online version contains supplementary material available at 10.1038/s41598-025-27358-2.

## Introduction

 Executive function (EF), which represents higher-order cognitive processes, is defined as the ability to select appropriate actions to achieve specific goals^1^. EFs are governed by the prefrontal cortex (PFC)^2^ and comprise three core components: inhibitory control, working memory, and cognitive flexibility^3^. Developing EFs during childhood is essential, as they form the foundation for self-regulation and social functioning. Furthermore, EFs are associated with academic achievement, obesity, depression, behavioral problems, and social interactions throughout childhood and adolescence^3–6^.

As children grow older, overall physical activity tends to decline, while sedentary behavior increases^7^. Between 2007 and 2016, the average sedentary time among U.S. children increased by 1.1 h^8^. Over 70% of the time children spend in school involves sedentary behavior^9^. The adverse effects of sedentary behavior on mental, physical, and cognitive health are concerning^10^, highlighting the need for effective strategies to break up prolonged sitting^11,12^. The primary aim of the present study was to investigate whether a brief, acute bout of light-intensity exercise can improve inhibitory control in children. We hypothesized that children would show improved inhibitory control following short-duration, light-intensity exercise. Previous systematic reviews and meta-analyses have demonstrated that physical exercise enhances EFs across the lifespan, from childhood to older adulthood^13–16^. These benefits can arise from both acute and chronic exercise. Acute exercise may transiently alter arousal, neurotransmitter levels, steroid hormone levels, cerebral blood flow, and neural efficiency^17–19^. In contrast, chronic exercise promotes structural and functional brain adaptations over time^20,21^, fostering long-term cognitive development. While chronic exercise interventions are essential for long-term cognitive development, acute exercise offers immediate but short-lived cognitive and emotional benefits that may help enhance learning readiness when incorporated at appropriate times in daily settings.

In studies focusing on children, a meta-analysis of 36 randomized controlled trials reported that both acute and chronic exercise improved inhibitory control and cognitive flexibility with small effect sizes, and working memory with a medium effect size^22^. However, a recent meta-analysis reported that the overall effects of chronic exercise in general on inhibitory control in children and adolescents were relatively small, suggesting the potential benefit of goal-directed physical activity interventions designed to enhance executive function^23^. These interventions primarily involved moderate-to-vigorous physical activities such as running, cycling, walking, and sports. However, these activities can be challenging to implement routinely in school settings because they require large spaces (e.g., outdoors and gymnasiums) and additional preparation time (e.g., clothing changes and transition periods).

Feasibility studies have highlighted the importance of intervention programs that can be delivered within the classroom, implemented in short durations (1–5 min), involve active teacher participation, and are well-accepted by users^24–26^. Several trials have evaluated short-duration exercise interventions in children. One randomized controlled trial involving 9–10-year-olds showed that an acute 5-min bout of moderate-to-vigorous rope-skipping enhanced EFs^27^, while another trial with 11–14-year-olds reported improvements in EFs following acute, 5-min, classroom-based, moderate-intensity activity breaks^28^. In terms of feasibility and acceptability, brief (≤ 5-min), light-intensity activities (e.g., stretching, light gymnastics, yoga) appear promising for regular implementation in school environments.

Although the number of studies is limited, some studies have demonstrated that light-intensity exercise can enhance inhibitory control. Inhibitory control refers to the ability to regulate attention, behavior, cognition, and emotions to override strong internal impulses or external stimuli in favor of more appropriate or goal-directed actions^3^. This function is critical in children’s school life, as it is associated with decreased off-task behavior in the classroom, improved academic performance, increased peer acceptance, and reduced risk of future delinquency^29–32^. In young adults, a single 10-min bout of light-intensity ergometer cycling improved inhibitory control and memory^33^.

Similarly, a 10-min acute light-intensity aerobic dance improved both inhibitory control and mood in older adults^34^. However, similar studies involving children are very limited. Mood enhancement is also particularly important because of the rising prevalence of mental health disorders in children and adolescents^35,36^. Exercise interventions have shown potential in improving mental health and overall well-being^37–40^. Furthermore, previous studies have demonstrated that exercise-induced arousal enhances inhibitory control^33^. Therefore, the secondary aim of this study was to investigate whether brief, acute bouts of light-intensity exercise can affect psychological mood in children.

To the best of our knowledge, no studies have examined the effects of short-duration, light-intensity exercise on inhibitory control and mood in children. Developing an intervention that is feasible across diverse school environments and capable of enhancing EF and mood may help mitigate the negative impacts of sedentary behavior and physical inactivity. Such a program could also strengthen the integration of structured activity breaks into the school curriculum.

## Methods

### Participants

The required sample size was calculated using G*Power 3.1.9.7, based on a 0.23-point effect size (partial eta squared = 0.05), an alpha level of 0.05, a power(1-β) of 0.95, and a correlation among repeated-measures of 0.8 for repeated-measures analysis of variance (ANOVA). The results indicated that 27 participants were required. The participants in this study included 31 healthy children (12.0 ± 1.1 years, 54.8% boys) residing in Tokyo and Saitama prefectures in Japan, ranging from the fifth grade of elementary school to the second grade of junior high school (age range 10–14 years). The inclusion criteria were as follows: (i) no history of mental or neurological disorders, (ii) no physician-imposed exercise restrictions, and (iii) no color vision deficiency. Participants were recruited using a snowball sampling method. Written informed assent was obtained from the children, and written informed consent was obtained from their parents prior to study participation. The present study was conducted with approval of the Waseda University Ethics Review Procedures Concerning Research with Human Subjects (approval number: 2021 − 344). All research procedures adhered to the Declaration of Helsinki and relevant ethical guidelines and regulations.

### Study design

**Fig. 1:**
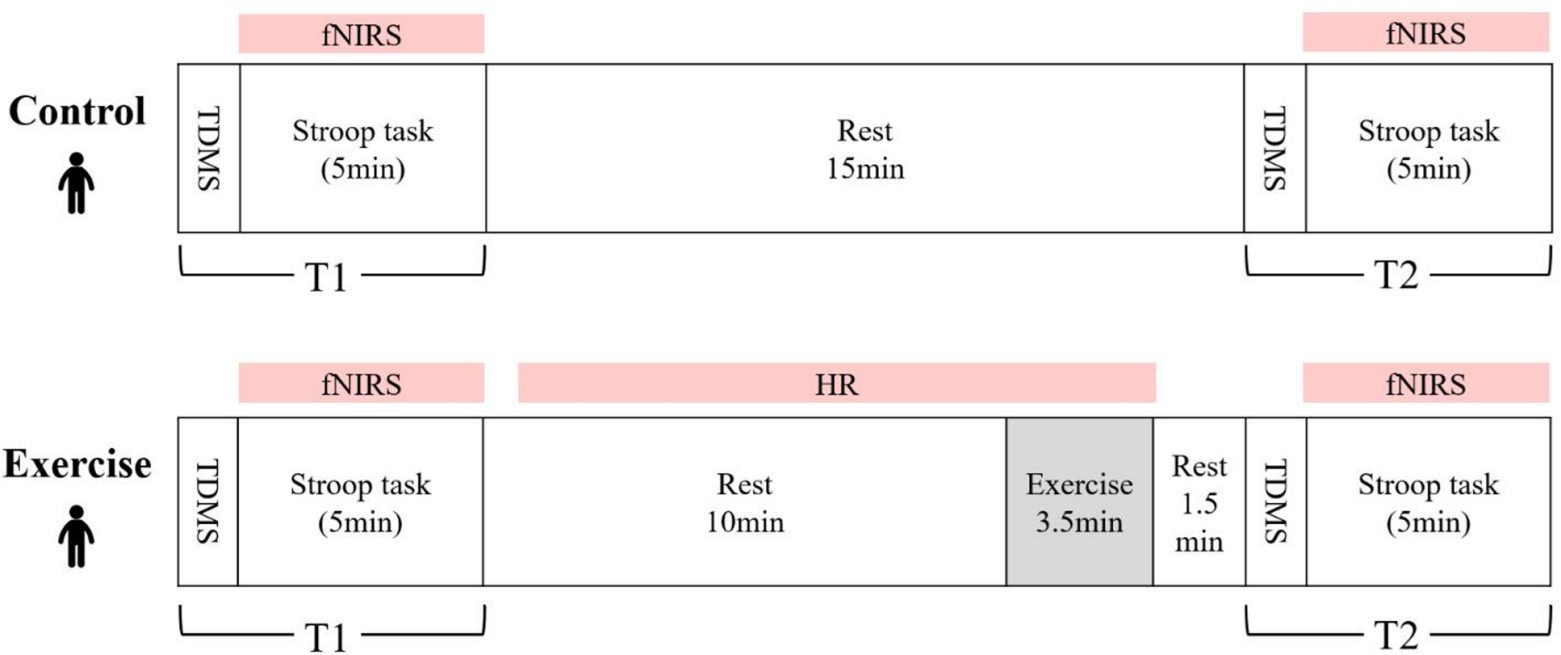
Experimental protocol. The order of conditions (Control, Exercise) was randomized. fNIRS, functional near-infrared spectroscopy measurements; HR, heart rate measurements; TDMS, Two-Dimensional Mood Scale; Stroop task, Color-word Stroop task; Rest, seated rest.

Using a crossover design, participants completed two experimental conditions (control and exercise) in a randomized order (Fig. 1). The experiment was conducted in a conference room at a public facility. Prior to the experiment, the sex, age, and dominant hand of each participant were recorded. Stature was measured to the nearest 0.1 cm (InLab, InBody Japan Inc., Tokyo, Japan), and body weight to the nearest 0.1 kg (BC-332 L, TANITA Corporation, Tokyo, Japan). Body mass index (BMI) was calculated by dividing the body weight (kg) by the stature (m) squared. The light-intensity exercise program, cognitive task (Color-word Stroop task; CWST), and psychological measures were explained to the participants. To minimize learning effects, participants completed a familiarization trial of the CWST for an average of approximately three minutes until their reaction times and error rates stabilized. These practice trials were conducted solely for familiarization and were not included in the analysis. After practice, a functional near-infrared spectroscopy (fNIRS) device (OEG-16 H; Spectratech Inc., Yokohama, Japan) was fitted to the participant’s head while seated, and sensor calibration was conducted. During the experiment, participants completed a psychological mood questionnaire and performed the CWST both before the break session (T1) and after the break session (T2) under each condition. In the control condition, the participants remained seated and at rest for 15 min between T1 and T2. In the exercise condition, participants remained seated and at rest for 10 min, performed a 3.5-min light-intensity exercise while watching a laptop monitor, and then rested for an additional 1.5 min. All participants completed one randomized condition, took a 15-min break, and then completed the other condition. The order of the two conditions was randomized across participants to balance potential order effects.

### Light-intensity exercise

The light-intensity exercise program lasted 3.5 min and included 6 distinct movements: dynamic stretching, static stretching with trunk rotation, single-leg balance, and hand dexterity exercises (Table 1). These activities were selected from a list of widely practiced exercises^41,42^ and were informed by a previous study^43^, that identified movement types potentially associated with PFC activation. This program is suitable for most children and can be easily implemented on-site, such as in a classroom, without the need for any equipment.

**Table 1:** Light-intensity exercise program performed in this study.

Event ID	1	2	3	4	5	6
Name	Elbow Circles	Side Bend	Trunk Twist	Hip Circles	Single Leg Balance	Thumb & Pinky
Position	Standing	Standing	Standing	Standing	Standing	Sitting
Type	DST	DST	SST	DST	Balance	Manual Dexterity
Reps / Time	10 each way	8 each way	2 each way	5 each way	15 s each leg	30 reps
Explanation	Keeping the fingers of both hands on the shoulders, rotate both elbows forward and backward.	Fold both hands above the head, extend them upward, and bend side to side alternately.	Fold both hands and extend them forward; twist the upper body side to side, holding each side for 5 s	Place hands on hips and rotate hips in circles, clockwise and counterclockwise	Place hands on hips, keep eyes open, lift one leg off the floor, and maintain balance.	Extend the thumb of one hand and the little finger of the other hand alternately.

DST, dynamic stretching; SST, static stretching.

### Heart rate measures

To quantify individual exercise intensity, heart rate (HR) was measured only during the exercise condition using a portable optical armband device (Polar Verity Sense; Polar Electro Oy, Kempele, Finland) worn on the non-dominant upper arm of each participant. In natural environments, the upper-arm-worn heart rate monitor has been reported to have a measurement accuracy comparable to that of commonly used chest-worn monitor (Pearson’s *r* = 0.91, *p* < 0.01)^44^. Resting heart rate (RHR) was calculated as the mean HR during a 5-min recording of quiet seated rest before the onset of exercise, following an initial 5-min period of quiet seated rest (no conversation or interaction in either period). HR during exercise was calculated as the mean HR across the exercise period. Individual exercise intensity was then calculated as a percentage of heart rate reserve (%HRR) using the Karvonen formula: %HRR = ([HR during exercise – RHR]/[HR max (220 – age) – RHR]) × 100^45,46^.

### Psychological measures

The Two-Dimensional Mood Scale (TDMS) was used to assess participants’ psychological mood status^47^. The TDMS includes 8 items (e.g., calm, irritated, lethargic, energetic, relaxed, tense, sluggish, and lively), rated on a 6-point Likert scale (0: Not at all to 5: Extremely). The TDMS was administered at T1 and T2 prior to the cognitive task (CWST) in each condition. Based on participant responses, pleasure, and arousal scores were calculated according to the TDMS guidelines.

### Cognitive performance measures

Inhibitory control, a component of executive function, was assessed using the Color-word Stroop task (CWST)^48^. Inhibitory control was defined as the capacity to control attention, behavior, thoughts, and/or emotions to override an internal impulse or external distraction and instead carry out a more appropriate or required action^3^. The CWST is widely recognized as a representative psychological tool for evaluating inhibitory processes. A Japanese-language CWST application for iOS (Stroop Test, Digital Standard Inc., Osaka, Japan) was installed on a tablet (iPad 10.2-inch, Apple Inc., Cupertino, CA, USA) and used for the measurements. Two CWST types were administered. In the neutral task, a colored circle (Red, Blue, Green, or Yellow) was displayed, and participants selected the correct button labeled “Red,” “Blue,” “Green,” or “Yellow” as quickly as possible. The labels were not color-coded. In the incongruent task, a colored word (e.g., “Red,” “Blue,” “Green,” or “Yellow”) was shown in ink of a different color (e.g., the word “Blue” in yellow ink), and participants were instructed to select the button matching the ink color, not the word meaning. Participants completed 60 trials per condition at T1 and T2, and both the reaction time and error rate were recorded.

### fNIRS measures

**Fig. 2:**
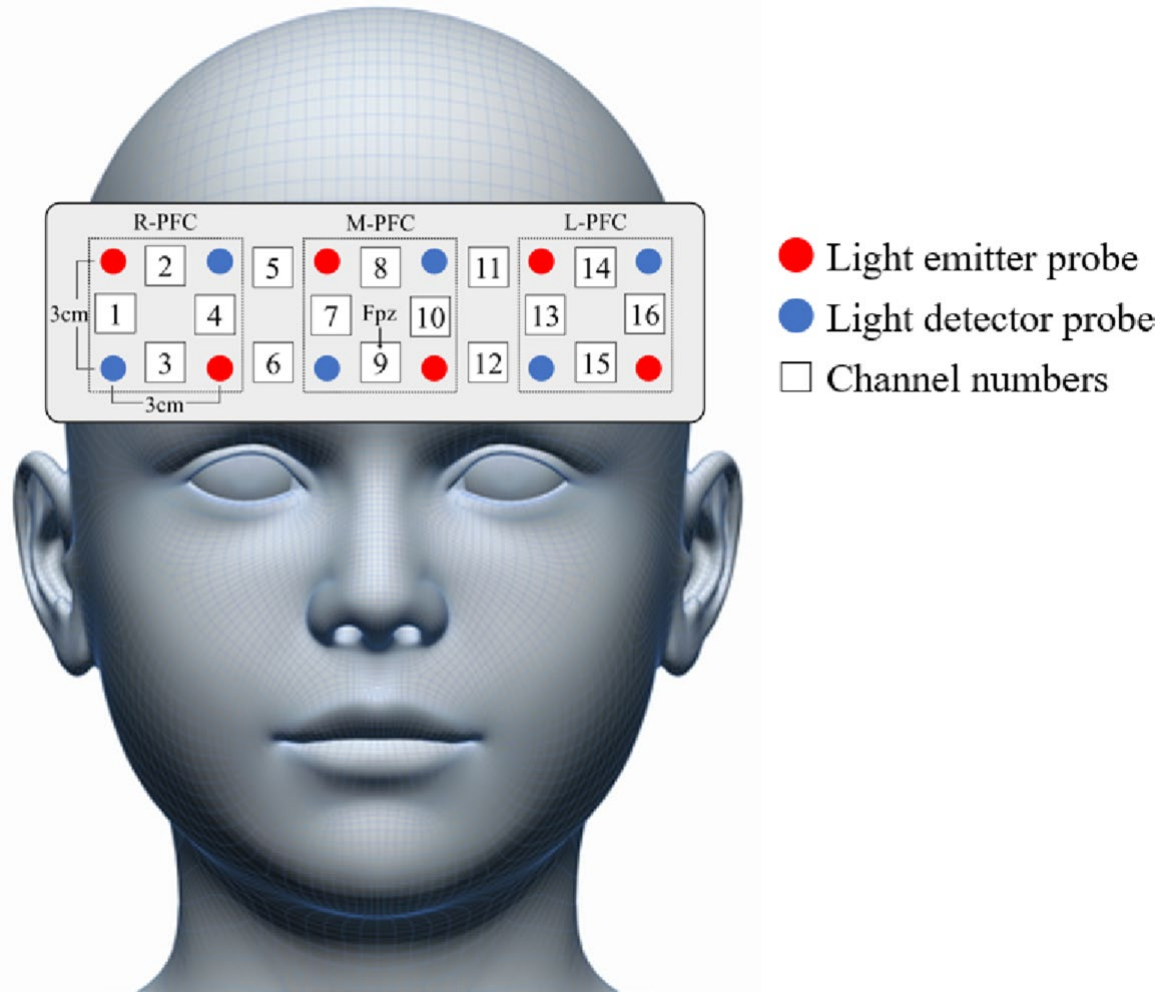
Locations of channels and attachment points for the probes in functional near-infrared spectroscopy. R-PFC, right prefrontal cortex; M-PFC, middle prefrontal cortex; L-PFC, left prefrontal cortex.

Functional near-infrared spectroscopy (fNIRS) is an optical imaging technique that assesses cerebral blood flow dynamics by emitting near-infrared light to the scalp. This light, which penetrates biological tissues such as the skin, bone, and muscle, enables the detection of hemodynamic changes in the brain. Specifically, fNIRS quantifies the fluctuations in the concentrations of oxygenated hemoglobin (oxy-Hb) and deoxygenated hemoglobin (deoxy-Hb) in the blood. Because near-infrared light can penetrate only up to approximately 2 cm from the scalp, this method is limited to measuring superficial cortical activity and does not detect changes in deeper brain structures. Although fNIRS has a lower spatial resolution than functional magnetic resonance imaging (fMRI), it offers advantages such as reduced sensitivity to motion artifacts and allows for the assessment of brain activity in more naturalistic settings, with fewer body position constraints. In this study, we used fNIRS to examine PFC hemodynamics during the CWST. The device consisted of 6 light-emitting sources and 6 detectors, forming 16 measurement channels. The near-infrared wavelengths were 770 and 840 nm, and the distance between each light source and detector was 3 cm. The probe placement was aligned according to the international 10–20 system, with the central lower row of sensors positioned over the participant’s Fpz location (Fig. 2). The sampling rate was set to 0.76 Hz (0.65535 s/1 sample), and changes in the concentrations of oxy-Hb, deoxy-Hb, and total hemoglobin in the PFC were recorded. The change in oxy-Hb is considered the most sensitive indicator of regional cerebral blood flow^49^. Therefore, we employed oxy-Hb as an indicator of cerebral blood flow in the PFC.

### fNIRS data processing

The fNIRS data were preprocessed using software installed on the measurement system. Initially, all recorded signals were processed using the hemodynamic separation method^50^ to eliminate increases in skin-blood flow caused by body movements^51^, which were not brain-derived signals. To remove artifacts related to respiration and cardiac pulsation, a high-pass filter at 0.01 Hz and a low-pass filter at 0.3 Hz were applied as bandpass filtering. The filtered data were exported as CSV files and converted to Excel format using Microsoft Excel for Microsoft 365 (Microsoft Corp., Redmond, WA, USA). For each event (neutral, incongruent), time point (T1 and T2), and condition (control and exercise), mean oxy-Hb values were calculated per channel between 0 and 5 s before the start of the CWST as the resting period, and during the CWST. Following a previous study^43,52^, channels were grouped into three regions: R-PFC (channels 1, 2, 3, and 4), M-PFC (channels 7, 8, 9, and 10), and L-PFC (channels 13, 14, 15, and 16). The mean oxy-Hb values at rest and during both the neutral and incongruent tasks were calculated for each region. Finally, the mean oxy-Hb values were converted into z-scores for each condition (control and exercise), time point (T1 and T2), and event (neutral, incongruent).

### Statistical analysis

Participants’ characteristics were analyzed using descriptive statistics. Resting HR and HR during exercise were compared using a paired t-test. At baseline (T1), differences in the CWST reaction time and error rate, pleasure level, and arousal level between the control and exercise conditions were analyzed using paired t-tests. Changes in the CWST reaction time, error rate, and TDMS scores were analyzed using a two-way repeated-measures ANOVA, with condition (control and exercise) and time (T1 and T2) as within-subject factors. When significant main effects or interactions were identified, post hoc comparisons were conducted using the Bonferroni correction. As the baseline pleasure level in the exercise condition was slightly lower than that in the control condition, we performed a one-way ANCOVA for pleasure, using baseline pleasure (T1) as a covariate, to examine whether baseline differences influenced post-intervention pleasure levels and to confirm the robustness of the main findings. In addition, one-way repeated-measures ANOVA was conducted for each region (R-PFC, M-PFC, and L-PFC) to compare oxy-Hb z-scores between the rest and the CWST periods. Throughout this manuscript, all reported η² values represent partial eta squared. For the variables with significant effects in ANOVAs, we examined the normality assumption using the Shapiro–Wilk test. For variables that did not meet the assumption (e.g., reaction time in the incongruent task), we additionally performed non-parametric sensitivity analyses (Friedman and Wilcoxon signed-rank tests) to confirm the robustness of the findings. Spearman’s rank correlation analyses were used to examine associations between changes in the CWST reaction time of the incongruent task (⊿reaction time) and changes in psychological mood (⊿pleasure level, ⊿arousal level) from T1 to T2, as well as changes in oxy-Hb z-scores (⊿oxy-Hb z-score) between the rest and the CWST periods, because some variables did not meet the assumption of normality. *P*-values for the correlation analyses were adjusted for multiple comparisons using the Benjamini–Hochberg procedure. Statistical significance was set at *P* < 0.05. All analyses were performed using IBM SPSS (version 29.0; SPSS Inc., Armonk, NY, USA).

## Results

Table 2 summarizes the characteristics of the participants. A total of 31 participants (17 boys, 53.1%, age range, 10–14 years) were included in the present study. The mean age was 12.0 ± 1.1 year (Boy, 12.6 ± 0.9; Girl, 11.2 ± 0.8), BMI was 19.4 ± 2.7 kg/m² (Boy, 20.0 ± 2.5 kg/m²; Girl, 18.7 ± 2.8 kg/m²).

**Table 2:** Participants’ characteristics.

	All Participants		Boy Participants		Girl Participants	
	Mean	SD	Mean	SD	Mean	SD
Age, years	12.0	1.1	12.6	0.9	11.2	0.8
BMI, kg/m^2^	19.4	2.7	20.0	2.5	18.7	2.8
Resting HR, bpm	78	12	73	10	84	12
HR during exercise, bpm	91	10	89	9	95	11
Exercise intensity, % HRR	10.1	4.1	11.5	3.9	8.4	3.7

BMI, body mass index; HR, heart rate; HRR, heart rate reserve; SD, standard deviation.

### Exercise intensity

In the exercise condition, the mean HR during exercise (91 ± 10 bpm) was significantly higher than the mean resting HR (78 ± 12 bpm) (t [30] = 12.88, *P* < 0.001, d = 2.31), corresponding to a 10.1 ± 4.1% HRR. Additionally, all participants exhibited < 30% HRR (individual range: 3.1–19.9%). Consequently, the exercise program implemented in this experiment was classified as very light-intensity^53^.

### Cognitive performance

At baseline (T1), no significant differences were observed between the control and exercise conditions in terms of the CWST reaction time and error rate (reaction time in the neutral task: t(30) = − 0.13, *P* = 0.896; reaction time in the incongruent task: t(30) = − 1.03, *P* = 0.311; error rate in the neutral task: t(30) = 1.29, *P* = 0.207; error rate in the incongruent task: t(30) = 0.09, *P* = 0.931).

**Fig. 3:**
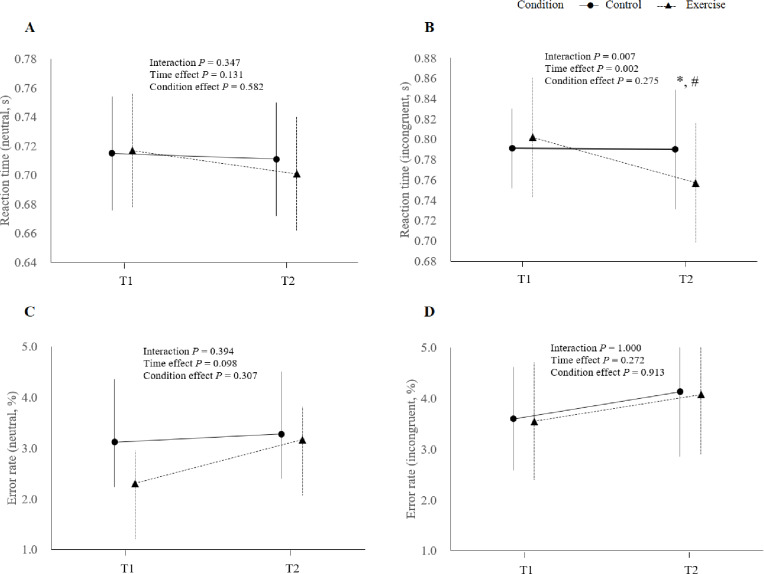
Changes in reaction time and error rate in the CWST for reaction times of neutral task (**A**), reaction times of the incongruent task (**B**), error rates of neutral task (**C**), error rates of the incongruent task (**D**). Error bars represent standard errors. The P values shown are the results of two-way ANOVA with time and condition as factors. * *P* < 0.001 vs. T1, # *P* < 0.05 vs. control condition.

Short-duration, light-intensity exercise was associated with a significant decrease in reaction time in the incongruent task of the CWST. The CWST results are shown in Fig. 3 and Supplementary Table S1. For the neutral task, the condition × time interaction was not significant (F [1,30] = 0.913, *P* = 0.347), and there were no main effects of condition (F [1,30] = 0.31, *P* = 0.582) or time (F [1,30] = 2.41, *P* = 0.131). In contrast, for the incongruent task, a significant condition × time interaction was observed (F [1,30] = 8.33, *P* = 0.007, η² = 0.22). At T1, there was no significant difference in reaction time between conditions (*P* = 0.311), whereas, at T2, the exercise condition demonstrated significantly shorter reaction times than the control condition (mean difference = 0.045 s, SE = 0.010, *P* = 0.001). Sensitivity analyses for the data that violated the normality assumption confirmed the robustness of these findings (Supplementary Table S3). For error rates, no significant main effects or interactions were identified.

### Psychological mood

At baseline (T1), no significant differences were observed between the control and exercise conditions in terms of psychological mood, including pleasure level (t(30) = 1.85, *P* = 0.074) and arousal level (t(30) = − 0.51, *P* = 0.614).

**Fig. 4:**
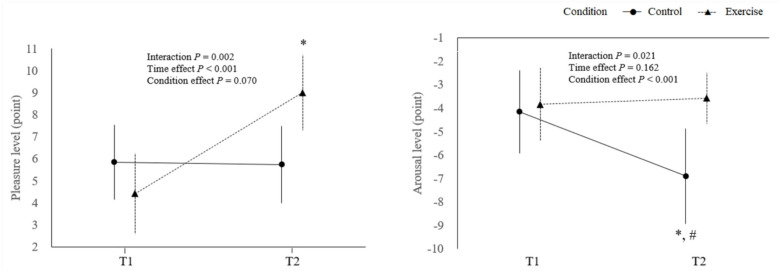
Changes in psychological mood for pleasure level (**A**), arousal level (**B**). Error bars represent standard errors. The P values shown are the results of two-way ANOVA with time and condition as factors. Pleasure: * *P* < 0.001 vs. T1: Arousal: * *P* < 0.05 vs. T1, # *P* < 0.01 vs. control condition.

Brief, light-intensity exercise increased pleasure level and prevented the decline in arousal observed in the control condition. The TDMS results are summarized in Fig. 4 and Supplementary Table S2. For pleasure level, a significant condition × time interaction was found (F [1,30] = 11.74, *P* = 0.002, η² = 0.28). At T1, there was no significant difference between the conditions (*P* = 0.074), whereas, at T2, the exercise condition showed significantly higher pleasure level than the control condition (mean difference = 3.26, SE = 0.91, *P* = 0.001). There was no significant main effect of condition (*P* = 0.070). Although there was no statistically significant difference in pleasure levels between the two conditions at T1, the baseline pleasure score in the exercise condition was approximately 1.4 points lower than that in the control condition. Therefore, as a sensitivity analysis, we performed an ANCOVA using baseline pleasure (T1) as a covariate to adjust for this difference. The analysis revealed that post-intervention pleasure was significantly higher in the exercise condition compared with the control condition (F [1,59] = 13.17, *P* < 0.001), indicating that the observed improvement in pleasure after exercise was independent of baseline differences. For arousal level, a significant condition × time interaction was found (F [1,30] = 5.91, *P* = 0.021, η² = 0.165). At T1, there was no significant difference between the conditions (*P* = 0.614). At T2, arousal was maintained in the exercise condition but declined in the control condition, resulting in lower arousal in the control condition (mean difference = -3.32, SE = 0.92, *P* = 0.001). There was no significant main effect of time (*P* = 0.162).

### Hemodynamic changes in the PFC

Overall, no consistent pattern of PFC activation emerged across regions. For the incongruent task of the CWST, PFC oxy-Hb z-score increased after the intervention in the control condition, whereas no significant changes were observed in the exercise condition. The relative changes in oxy-Hb levels are presented in Table 3. In the control condition, oxy-Hb z-scores significantly increased in the L-PFC during the incongruent task at both T1(F [1,30] = 4.75, *P* = 0.037, η² = 0.14) and T2(F [1,30] = 5.91, *P* = 0.021, η² = 0.17), and in the R-PFC at T2(F [1,30] = 8.21, *P* = 0.008, η² = 0.22). In the exercise condition, oxy-Hb z-scores significantly increased during the neutral task at T1 in the R-PFC (F [1,30] = 8.81, *P* = 0.006, η² = 0.23), M-PFC (F [1,30] = 7.56, *P* = 0.010, η² = 0.20), and L-PFC (F [1,30] = 5.24, *P* = 0.029, η² = 0.15). At T2, a significant decrease in the oxy-Hb z-score was observed only in the R-PFC during the neutral task (F [1,30] = 4.31, *P* = 0.047, η² = 0.13). Sensitivity analyses for the data that violated the normality assumption confirmed the robustness of these findings (Supplementary Table S3). No significant increases were found during the incongruent task at either T1 or T2.

**Table 3:** Hemodynamic changes in the prefrontal cortex before and during the CWST.

			oxy-Hb (mM・mm)				oxy-Hb z-score						
			Pre		Cog		Pre		Cog		F	*P*	η2
			Mean	SE	Mean	SE	Mean	SE	Mean	SE			
(A) Control condition													
T1	Neutral	R-PFC	-0.06	0.04	0.06	0.04	-0.25	0.18	0.25	0.18	2.68	0.112	0.08
		M-PFC	-0.01	0.04	0.05	0.05	-0.13	0.18	0.13	0.19	0.67	0.421	0.02
		L-PFC	-0.04	0.04	0.02	0.04	-0.16	0.20	0.16	0.16	1.56	0.222	0.05
	Incongruent	R-PFC	-0.04	0.05	0.01	0.04	-0.13	0.20	0.13	0.16	1.19	0.284	0.04
		M-PFC	-0.04	0.06	0.03	0.04	-0.25	0.31	0.25	0.31	0.61	0.439	0.02
		L-PFC	-0.09	0.05	0.03	0.03	-0.26	0.21	0.26	0.14	4.75	0.037*	0.14
T2	Neutral	R-PFC	0.16	0.21	-0.03	0.05	-0.16	0.25	-0.16	0.06	1.76	0.195	0.06
		M-PFC	0.09	0.18	0.05	0.09	0.06	0.23	-0.06	0.11	0.23	0.632	0.01
		L-PFC	0.03	0.18	0.04	0.05	0.02	0.25	-0.02	0.07	0.02	0.883	0.00
	Incongruent	R-PFC	-0.09	0.08	0.18	0.07	-0.29	0.19	0.29	0.16	8.21	0.008*	0.22
		M-PFC	-0.02	0.12	0.13	0.07	-0.14	0.22	0.14	0.13	1.46	0.236	0.05
		L-PFC	-0.07	0.08	0.21	0.06	-0.30	0.20	0.30	0.14	5.91	0.021*	0.17
(B) Exercise condition													
T1	Neutral	R-PFC	-0.08	0.04	0.05	0.03	-0.42	0.18	0.42	0.16	8.81	0.006*	0.23
		M-PFC	-0.09	0.05	0.08	0.03	-0.41	0.19	0.41	0.15	7.56	0.010*	0.20
		L-PFC	-0.07	0.04	0.06	0.04	-0.35	0.17	0.35	0.17	5.24	0.029*	0.15
	Incongruent	R-PFC	0.02	0.05	0.03	0.04	-0.10	0.20	0.10	0.17	0.41	0.528	0.01
		M-PFC	0.03	0.06	0.05	0.05	-0.04	0.19	0.04	0.17	0.05	0.820	0.00
		L-PFC	0.01	0.06	0.01	0.03	-0.03	0.22	0.03	0.13	0.05	0.825	0.00
T2	Neutral	R-PFC	0.42	0.19	0.02	0.04	0.24	0.24	-0.24	0.06	4.31	0.047*	0.13
		M-PFC	0.45	0.23	0.10	0.10	0.14	0.23	-0.14	0.11	2.10	0.158	0.07
		L-PFC	0.19	0.17	0.06	0.05	0.08	0.25	-0.08	0.07	0.58	0.452	0.02
	Incongruent	R-PFC	0.15	0.11	-0.02	0.03	0.16	0.25	-0.16	0.07	1.61	0.215	0.05
		M-PFC	0.33	0.28	-0.01	0.04	0.16	0.25	-0.16	0.04	1.20	0.282	0.04
		L-PFC	0.16	0.13	-0.02	0.04	0.14	0.24	-0.15	0.08	0.97	0.332	0.03

CWST, Color-word Stroop task; pre, before the CWST; cog, during the CWST, oxy-Hb, oxygenated hemoglobin; SD, standard deviation; SE, standard error. R-PFC, right prefrontal cortex; M-PFC, middle prefrontal cortex; L-PFC, left prefrontal cortex.

### Correlation between changes in reaction time, psychological mood, and PFC oxy-Hb z-score

Weak correlations were observed between ⊿reaction time and ⊿pleasure level, and between ⊿reaction time and ⊿oxy-Hb z-scores in the R-PFC and L-PFC. However, none of these associations remained statistically significant after adjusting for multiple comparisons using the Benjamini–Hochberg procedure (Table 4).

**Table 4:** Correlation between the CWST ⊿reaction time of the incongruent task and changes in psychological mood and PFC ⊿oxy-Hb z-score.

		⊿pleasure level	⊿arousal level	*R*-PFC ⊿oxy-Hb z-score	M-PFC ⊿oxy-Hb z-score	L-PFC ⊿oxy-Hb z-score
(A) ⊿reaction time (incongruent task)						
⊿reaction time (incongruent)	r	-0.30	0.10	0.25	0.10	0.29
	Adjusted *P*	0.060	0.437	0.083	0.437	0.060
(B) ⊿pleasure level						
⊿pleasure level	r	-0.30	0.25	-0.23	-0.15	-0.18
	Adjusted *P*	0.085	0.118	0.118	0.205	0.236

*n* = 62 for all correlation analyses. CWST, Color-word Stroop task; oxy-Hb, oxygenated hemoglobin; R-PFC, right prefrontal cortex; M-PFC, middle prefrontal cortex; L-PFC, left prefrontal cortex. *P*-values were adjusted for multiple comparisons using the Benjamini–Hochberg procedure.

## Discussion

This study investigated whether acute short-duration, light-intensity exercise affects EFs and psychological mood states. To the best of our knowledge, this is the first study to demonstrate that short-duration (3.5-min), light-intensity (10.1% HRR) acute exercise significantly enhanced inhibitory control and pleasure level among children. The reaction time of the CWST incongruent task in the exercise condition significantly shortened, without a speed–accuracy trade-off. After 15 min of prolonged sitting, the arousal level significantly decreased, whereas implementing brief light-intensity exercise preserved arousal and increased pleasure levels.

In this study, following light-intensity exercise, participants’ pleasure level was significantly enhanced, whereas their arousal levels remained unchanged. In a previous study, after 10 min of acute open-skill (badminton) or closed-skill (running) vigorous-intensity exercises, participants’ arousal levels significantly increased, while pleasure level did not^52^. After 10 min of acute light-intensity ergometer cycling in young and older adults, the arousal level was enhanced^33,54^, but the pleasure level remained unaffected. Conversely, after 10 min of acute slow-tempo aerobic exercise in older adults, both arousal and pleasure levels were enhanced^34^. Based on these findings, the 3.5-min light-intensity exercise employed in this study may have been insufficient in intensity or duration to increase the arousal level, whereas it may have been adequate to increase the pleasure level. As the arousal level did not increase but was maintained, including acute brief light-intensity exercise during prolonged sitting may be beneficial for maintaining arousal.

According to the reticular-activating hypofrontality (RAH) model, acute exercise enhances arousal, while EFs temporarily decline during exercise but recover afterward, leading to increased frontal lobe activity and enhanced EFs post-exercise^55^. However, the exercise in this study did not enhance arousal, as predicted by the RAH model, yet the reaction time improved. Several factors may contribute to this improvement. Although weak correlations were observed between the increased pleasure level after exercise and improvements in inhibitory control, none of these associations remained statistically significant after adjusting for multiple comparisons. A previous review article that primarily involved 15–30 min of exercise demonstrated that most individuals experience pleasure under the ventilatory or lactate threshold, and negative affect at supra-threshold intensities^56^. Despite engaging in similar light-intensity exercises, the pleasure level significantly increased after the slow-tempo aerobics and light-intensity program used in this study, whereas it did not increase after ergometer cycling. These findings suggest that to enhance pleasure level with short-duration exercise, variation in movement, such as in slow-tempo aerobics or the light-intensity program used here, is essential, as opposed to monotonous exercises like ergometer cycling.

Some components of the exercise program used in this study, such as dynamic stretching, single-leg balance, and finger dexterity, included elements of cognitively demanding coordinative exercise^43^. Budde et al. hypothesized that cognitively demanding exercise may pre-activate specific brain regions^57^. Previous studies have shown that acute coordinative exercise enhances executive functions and attentional performance, possibly through increased activation of brain regions involved in motor–cognitive integration and the modulation of neuroendocrine responses^57,58^. Therefore, the presence of coordinative elements in the exercise program may have partially contributed to the observed improvements in inhibitory control in this study. Another factor that may have contributed to the improvement in inhibitory control is that the low-intensity exercise adopted in this study may have been suitable for enhancing inhibitory control. Previous studies have shown that acute low- to moderate-intensity exercise can improve executive functions, including working memory and inhibitory control, whereas high-intensity exercise may impair these functions^18,59^. This effect may be mediated by beneficial neuroendocrine responses, such as the appropriate regulation of steroid hormones, and by avoiding excessive physiological stress associated with high-intensity exercise. These findings highlight the importance of carefully adjusting exercise intensity when designing physical activity interventions aimed at achieving cognitive benefits^60^.

Previous research has demonstrated that the L-PFC is activated during incongruent tasks compared with neutral tasks in the CWST^52,61^. Consistent with these findings, oxy-Hb z-scores in the L-PFC increased during both T1 and T2 of the incongruent task in this study. However, in the exercise condition, a significant increase in the oxy-Hb z-score was not observed for the incongruent tasks. At T1, oxy-Hb z-scores significantly increased across all regions (R-, M-, and L-PFC) prior to the intervention in the exercise condition. The oxy-Hb value may have been relatively low at baseline, which could have influenced the L-PFC z-score and contributed to the absence of a significant increase. One possible explanation for this unexpected pattern of PFC activation is its psychological nature. The pleasure state was over 1.2 points lower in the exercise condition at T1 compared to the control condition. Additionally, the HR monitor band was attached to the arm before the experiment began only in the exercise condition, which may have induced discomfort. Psychological states and the anticipation of upcoming exercise have been shown to influence cerebral blood flow in the prefrontal cortex^62,63^. Generally, when unpleasant emotions are elicited, the limbic system involved in emotion processing becomes activated. In turn, the PFC, responsible for cognitive regulation, becomes active to suppress limbic system activity and reduce negative affect^64^. Since this study involved children, whose PFCs are still developing, this regulatory process may not have been fully functional, contributing to the observed pattern. Despite this difference at T1, the data suggested that the effect did not persist at the T2 measurement, indicating no issue in comparing oxy-Hb at T2. Therefore, we proceeded with the analysis of oxy-Hb z-score changes between the control and exercise conditions at T2 (post-intervention).

In this study, reaction time during incongruent tasks significantly improved following the short-duration, light-intensity exercise intervention. In contrast, no significant change in the PFC oxy-Hb z-score during the incongruent task was observed under the exercise condition. Additionally, the improvement in reaction time for the incongruent task was weakly correlated with decreased ⊿oxy-Hb z-scores in the R-PFC and L-PFC. These results may suggest that short-duration, light-intensity exercise intervention could have enhanced neural efficiency, potentially enabling cognitive processing without further activation of the PFC. This phenomenon may align with the Neural Efficiency Hypothesis, which proposes that cognitive performance can improve without a corresponding increase in brain activation during task execution^65^. Our findings are consistent with a previous study showing improved CWST reaction time in the incongruent task following a 10-min bout of acute badminton, without significant changes in PFC oxy-Hb among young adults^52^. Another study reported that the CWST reaction time improved after 15 min of treadmill walking in older adults, accompanied by increased oxy-Hb in the M-PFC, whereas no oxy-Hb changes were observed in younger adults^66^. In the present study, reaction time improved without changes in oxy-Hb z-scores, which may be attributed to the younger age of the participants. However, another study found that a 15-min moderate-intensity acute ergometer exercise improved the CWST reaction time with increased oxy-Hb in the left PFC^67^. Acute high-intensity interval training and 10-min ergometer cycling have also been shown to activate the left-dorsolateral prefrontal cortex (left-DLPFC) and enhance inhibitory control in young adults^33,68^. Thus, previous findings remain inconsistent, likely due to methodological variations, including the specific PFC regions analyzed, exercise modality, intensity, duration, and participant age. Although we interpreted these findings as a potential indication of enhanced neural efficiency, this explanation should be considered with caution. Other factors, such as ceiling effects in task performance, inter-individual variability in hemodynamic responses, or slight inaccuracies in probe placement, might also explain the lack of significant oxy-Hb increases during the incongruent task in the exercise condition. Additionally, because some participants had smaller head circumferences than adults, we analyzed the data by grouping channels into the right, middle, and left PFC regions rather than examining each channel individually. This approach allowed for more stable data interpretation but may have limited the ability to detect more localized effects. Further research accounting for these factors is needed to clarify the relationship between exercise and neural efficiency.

The WHO recommends limiting sedentary time for children and adolescents^69^. Several national guidelines advise breaking up prolonged sedentary periods as frequently as possible^70,71^. In schools, where children spend most of their time, sedentary behavior accounts for over 70% of daily activity. The Sedentary Behaviour Research Network recommends interrupting prolonged sitting at least every 30 min for children aged 5–11 years and at least every hour for adolescents aged 12–18 years, incorporating activities of various intensities and durations (e.g., standing, stretching breaks, active lessons, and activity breaks)^72^. Research involving teachers and principals has identified time constraints as the most significant barrier to implementing movement-based interventions in schools, emphasizing the importance of academic integration and feasibility within short timeframes^24^. One prior study demonstrated that interrupting 80 min of prolonged sitting with four 3-min resistance activity sessions improved working memory in adolescents^11^. The present study also improved EFs, closely tied to academic performance, through activities that can be implemented on the spot and completed within a short-duration. Additionally, because the exercise format involves simple movements (e.g., standing stretches, balance tasks, and hand exercises), it may be particularly feasible to introduce in school environments. Short-duration, light-intensity interventions in schools may contribute to improving children’s readiness to learn in a single session. However, it is important to note that fostering long-term cognitive development requires habitual and repeated implementation. Recent studies have indicated that increased light-intensity physical activity supports obesity prevention, reduces systemic inflammation, and benefits cardiovascular health in children and adolescents^73–76^. Considering these additional health benefits, the brief, classroom-compatible, light-intensity exercise tested in this study could offer broader implications beyond cognitive and psychological outcomes, potentially contributing to comprehensive health promotion among children. Future research should further explore these wider health outcomes in addition to cognitive and mood effects.

The present study has several limitations. First, participants completed both conditions (rest and exercise) on the same day. Although this design allowed us to control for variability in participants’ daily schedules, it also represents a major limitation of the study because potential carryover effects cannot be completely ruled out. Considering the cultural and practical context in Japan, where most children aged 10–14 years are engaged in extracurricular activities such as sports or music clubs and attend cram schools after school or on weekends, conducting sessions on separate days would have complicated the control of the participants’ physical and cognitive states on the day of each experiment. Accordingly, we opted to conduct both conditions on the same day, using a national holiday that enabled control of the effects of extracurricular activities. Furthermore, due to the considerable individual differences in brain development among children in this age range, a within-subjects (crossover) design would better maintain internal validity by minimizing inter-individual variability compared with a between-subjects design. Additionally, to mitigate potential order effects, the order of the two conditions was randomized, and we found no significant baseline differences in the CWST reaction time, error rate, pleasure level, or arousal level between the two conditions. Even if present, residual learning effects, cognitive fatigue, or acute exercise–induced benefits (e.g., transient improvements in EF and mood) cannot be completely ruled out as potential carryover into the subsequent condition, particularly when the exercise condition preceded the control condition. To address this limitation more rigorously, future studies would benefit from including a sufficient washout period between sessions. Second, the age range of the participants was restricted to 10–14 years. Expanding the age range and increasing the sample size are necessary to examine whether similar effects are observed across children and adolescents of various ages. Third, participants were recruited using a snowball sampling method, which may have introduced selection bias. Additionally, because the participants lived in urban areas of Tokyo and Saitama, further research including children from rural areas is needed to enhance generalizability to Japanese school-aged children. Fourth, this study focused on the acute effects of exercise on PFC activation and mood level. Future research should investigate the impact of habitual short-duration, light-intensity exercise on EFs and mood. Fifth, only PFC hemodynamics and mood were measured. Additional potentially relevant factors, such as autonomic nervous system activity, should also be assessed to better understand the mechanisms underlying EF improvements. Sixth, this study targeted the effects of short-duration, light-intensity exercise on inhibitory control. Further research is required to explore how this type of exercise influences other EF components, such as working memory and cognitive flexibility. Lastly, the intervention consisted of dynamic stretching, static stretching with twist movements, balance exercises, and hand exercises, all of which have been suggested to activate the PFC. Although these exercises likely contributed to EF enhancement, future studies should compare their effects with those of monotonous static stretching, moderate aerobic exercise, or high-intensity interval training, and learning-related tasks involving psychological load of the same duration to better control for nonspecific effects^77,78^. Despite these limitations, the findings offer a valuable, socially applicable strategy for enhancing children’s EF and mood, while also promoting physical activity and reducing sedentary behavior in classroom environments.

## Conclusion

Acute 3.5-min light-intensity exercise enhanced inhibitory control and pleasure level while maintaining arousal level among children. These findings suggest that such simple, classroom-compatible activities may contribute to cognitive and emotional support in educational settings.

## Supplementary Information

Below is the link to the electronic supplementary material.

Supplementary Material 1

## Data Availability

The datasets generated and/or analyzed in the current study are not publicly available due to ethical considerations; however, they are available from the corresponding author upon reasonable request.
