# Supplementary material for: Acute 3.5-minute light-intensity exercise enhances executive function and psychological mood in children
Source: Sci Rep. 2025 Dec 5;15:43198. doi: 10.1038/s41598-025-27358-2 (PMC12680618; doi:10.1038/s41598-025-27358-2)
Supplement: Supplementary file 1 — Supplementary Material 1 [file 41598_2025_27358_MOESM1_ESM.docx]

S1 Table. Changes in reaction time and error rate in CWST

|  | **Control condition** | |  | **Exercise condition** | |  | **Condition** | | | |  | **Time** | | | |  | **Interaction** | | | |
| --- | --- | --- | --- | --- | --- | --- | --- | --- | --- | --- | --- | --- | --- | --- | --- | --- | --- | --- | --- | --- |
|  | **Mean** | **SE** |  | **Mean** | **SE** |  | **F** | **df** | **η2** | ***P*** |  | **F** | **df** | **η2** | ***P*** |  | **F** | **df** | **η2** | ***P*** |
| **Reaction time (neutral, s)** |  |  |  |  |  |  |  |  |  |  |  |  |  |  |  |  |  |  |  |  |
| T1 | 0.715 | 0.02 |  | 0.717 | 0.02 |  | 0.31 | 30 | 0.01 | 0.582 |  | 2.41 | 30 | 0.07 | 0.131 |  | 0.91 | 30 | 0.03 | 0.347 |
| T2 | 0.711 | 0.02 |  | 0.701 | 0.02 |  |  |  |  |  |  |  |  |  |  |  |  |  |  |  |
| **Reaction time (incongruent, s)** |  |  |  |  |  |  |  |  |  |  |  |  |  |  |  |  |  |  |  |  |
| T1 | 0.791 | 0.02 |  | 0.802 | 0.03 |  | 1.24 | 30 | 0.04 | 0.275 |  | 11.82 | 30 | 0.28 | 0.002 |  | 8.33 | 30 | 0.22 | 0.007 |
| T2 | 0.790 | 0.03 |  | 0.757 | 0.03 |  |  |  |  |  |  |  |  |  |  |  |  |  |  |  |
| **Error rate (neutral, %)** |  |  |  |  |  |  |  |  |  |  |  |  |  |  |  |  |  |  |  |  |
| T1 | 3.12 | 0.63 |  | 2.31 | 0.33 |  | 1.08 | 30 | 0.04 | 0.307 |  | 2.91 | 30 | 0.09 | 0.098 |  | 0.75 | 30 | 0.02 | 0.394 |
| T2 | 3.28 | 0.45 |  | 3.17 | 0.56 |  |  |  |  |  |  |  |  |  |  |  |  |  |  |  |
| **Error rate (incongruent, %)** |  |  |  |  |  |  |  |  |  |  |  |  |  |  |  |  |  |  |  |  |
| T1 | 3.60 | 0.52 |  | 3.55 | 0.59 |  | 0.01 | 30 | 0.00 | 0.913 |  | 1.25 | 30 | 0.04 | 0.272 |  | 0.00 | 30 | 0.00 | 1.000 |
| T2 | 4.13 | 0.65 |  | 4.08 | 0.60 |  |  |  |  |  |  |  |  |  |  |  |  |  |  |  |

CWST, Color-word Stroop task; SE, Standard error

S2 Table. Changes in psychological mood level

|  | **Control condition** | |  | **Exercise condition** | |  | **Condition** | | | |  | **Time** | | | |  | **Interaction** | | | |
| --- | --- | --- | --- | --- | --- | --- | --- | --- | --- | --- | --- | --- | --- | --- | --- | --- | --- | --- | --- | --- |
|  | **Mean** | **SE** |  | **Mean** | **SE** |  | **F** | **df** | **η2** | ***P*** |  | **F** | **df** | **η2** | ***P*** |  | **F** | **df** | **η2** | ***P*** |
| **Pleasure** |  |  |  |  |  |  |  |  |  |  |  |  |  |  |  |  |  |  |  |  |
| T1 | 5.84 | 0.87 |  | 4.42 | 0.93 |  | 3.53 | 30 | 0.11 | 0.070 |  | 15.90 | 30 | 0.35 | < 0.001 |  | 11.74 | 30 | 0.28 | 0.002 |
| T2 | 5.74 | 0.89 |  | 9.00 | 0.87 |  |  |  |  |  |  |  |  |  |  |  |  |  |  |  |
| **Arousal** |  |  |  |  |  |  |  |  |  |  |  |  |  |  |  |  |  |  |  |  |
| T1 | -4.16 | 0.90 |  | -3.84 | 0.80 |  | 13.50 | 30 | 0.31 | < 0.001 |  | 2.05 | 30 | 0.06 | 0.162 |  | 5.91 | 30 | 0.17 | 0.021 |
| T2 | -6.90 | 1.04 |  | -3.58 | 0.56 |  |  |  |  |  |  |  |  |  |  |  |  |  |  |  |

SE, Standard error

S3 Table. Non-parametric sensitivity analyses for variables violating the normality assumption

| Category | Comparison | Test | *P* | Results | Reference Table |
| --- | --- | --- | --- | --- | --- |
| CWST reaction time | Control-T1, Control-T2, Exercise-T1, Exercise-T2 | Friedman χ² (3) = 18.90 | < 0.001 | Significant difference across time points. | Table 4,  S1 Table |
|  | Control-T2, Exercise-T2 | Wilcoxon Z = -2.54 | 0.011 | Exercise-T2 was significantly shorter than Control-T2. |  |
|  | Exercise-T1, Exercise-T2 | Wilcoxon Z = -3.82 | < 0.001 | Exercise-T2 was significantly shorter than Exercise-T1. |  |
| Hemodynamics change (oxy-Hb z-score) | Control, T2, Incongruent, R-PFC, Pre vs. Cog | Wilcoxon Z = -2.78 | 0.005 | Cog was significantly higher than Pre. | Table 3 |
|  | Control, T2, Incongruent, L-PFC, Pre vs. Cog | Wilcoxon Z = -2.15 | 0.032 | Cog was significantly higher than Pre. |  |
|  | Exercise, T1, Neutral, M-PFC, Pre vs. Cog | Wilcoxon Z = -2.34 | 0.019 | Cog was significantly higher than Pre. |  |
|  | Exercise, T2, Neutral, R-PFC, Pre vs. Cog | Wilcoxon Z = -1.99 | 0.047 | Cog was significantly higher than Pre. |  |

The normality assumption was examined for all variables that showed significant effects in this study. To confirm the robustness of the findings, non-parametric sensitivity analyses were conducted for variables that violated the assumption. Variables not listed in this table all met the normality assumption.
